# On Eczema of the Fingers and Its Treatment

**Published:** 1882-02

**Authors:** J. Magee Finny


### SELECTIONS.

### ON ECZEMA OF THE FINGERS AND ITS TREATMENT.

DR. J. MAGEE FINNY, in the *Dublin Jour. of Med. Science.*

In general, as in hospital practice, hardly a day passes without the physician being called upon to treat cases of eczema.

Of the protean varieties of eczema it is not my intention to speak. I desire to address myself to the disease as it presents itself on the fingers and in some other special localities, because these varieties are passed over in a few, generally short, sentences in the treatises on skin diseases.

The most prominent symptom of eczema of the fingers is pruritus, and this itching often first calls attention to the eruption. It is present at every stage; at first it is more of a tingling sensation, relieved by firm pressure, but in the stage of infiltration and squamation, it cannot be allayed by less than the liberal use of the nails. This pruritus is common in every variety of eczema, and so severe is it at times, when the disease is situated in the scrotum or in the eyebrows, I have known the scratching to be carried to such a degree as to remove every hair from the affected part.

Eczema digitorum is very chronic in its course, and, with seeming improvements it recurs again and again with increased severity. Hebra thus writes of it: "Of all varieties of eczema the most obstinate and difficult to cure is that which attacks the fingers, and here it is also peculiarly liable to recur."

Of the many other varieties of eczema and dermatitis engaging the hand and fingers, which are caused by mechanical irritants attendant on several trades and occupations, I wish to refer to only one—a very common one in winter and spring, which occurs in nurses and those who are engaged much in washing. It is extremely painful, rendering the hands almost useless, and yet it is very readily cured. I mean the inflammation attacking the distal phalanges close to the nails. Deep and wide fissures ensue so that the fingers are rendered useless for needle work, or indeed for any other work, and they are so painful that often rest at night is broken and disturbed. Several methods of treatment have been suggested, but there is nothing which I have found to come up to frequently coating the fissures, and the ends of the fingers generally, with a solution of gutta-percha dissolved in chloroform. Painful at the moment of application, it soon gives the greatest relief, and has the advantage of not requiring the patient to give up her work, even for a time, and of not heating the finger, as india-rubber finger-stalls are likely to do. I have employed collodion flexile in similar cases, but it has not answered at all as well as the chloroform solution.

Excluding mechanical and chemical irritants and venous obstruction, as causes of eczema, dyspepsia, worry, gout and scrofula are recognized predisponents. In looking through the notes of my cases of *localized* eczema digitorum, I find the majority occurred in persons over thirty years of age, and that no case in children presented itself to my notice. Those under thirty generally conformed to the type of scrofula, while many of those over thirty exhibited characters of gout. At the time of the rash it was noticeable that in neither class was there any pronounced type of the respective diathesis present, as if the *materies morbi* were not sufficient to cause either a gouty paroxysm or pulmonary congestion, but expended its force on the skin. However that may have been, it was a great help to the treatment to recognize either scrofula or gout as factors in the case, and it is a point, I consider, which should not be overlooked in the management of such cases. There is another class of patients in which I have noticed eczema to be of frequent occurrence which does not come exactly under either of the above heads. It is that of overworked nervous persons with much anxiety of mind, such as governesses or mothers of large families. In these cases the benefit which has followed upon the use of pure phosphorus was at once gratifying and remarkable.

There are very many points in common which render the diagnosis difficult, and particularly so when scabies attacks adults of strict personal cleanliness, and who are in the habit of frequently washing their hands. In such cases one never meets with the pronounced gross features of scabies of the "great unwashed." The situation being on the fingers and often the interdigital spaces, the intolerable itching augmented by heat and the existence of vesicles, makes the resemblance very strong. In distinguishing between them it is well to recognize the greater frequency of scabies at all ages, and especially in the young, in every station of life, as compared with eczema digitorum.

Vesicles are present in both eczema and scabies, but in eczema they are small, uniform in size, and clustered—involving a large part of the finger; the skin, moreover, becomes infiltrated and thickened with various cracks over the joints, and the seat of the disease is accurately defined; while in scabies vesicles occur only here and there on several fingers, wherever the young acari lie, and beside the burrows of the females, and hence they are few in number and discrete; again, they are found on the palm and wrist at the head of the ulna, as well as between the fingers. Moreover, in scabies, in certain irritable skins, a number of secondary eruptions—erythema, pustules, bullæ, and scabs—may be detected about the hand, on the wrist and palm, and extending up the arm. The very irritation differs, for the itching of scabies is stated to be less unpleasant than that of eczema, and to be free from the smarting and tingling of the latter.

Should the eczema digitorum be but part of general eczema, with itching in different parts of the body, it will not follow the course of scabies, for the latter shows itself wherever the insect is carried by the finger-nails, or where the clothes by their tightness foster the acarus—thus at the waist and under the breast in women, on the shoulders and on the dorsum of the penis in men.

A matter of care in every case, the diagnosis becomes a matter of considerable difficulty when an original scabies has been so persistently and energetically treated by inunctions of sulphur that a true irritative eczema is superinduced, and not being recognized, but looked upon as a part of the original complaint, is still further aggravated by increased activity of treatment.

Under such circumstances—and they are by no means as rare as those who see little of skin diseases might imagine—an accurate diagnosis can be arrived at only when soothing treatment has been employed, and the sulphur totally suspended for a few days. The scabies may then be found cured, and the eczema, the result of too strong and too vigorous medication, will be sufficiently defined to be recognized. Should doubt still exist, the discovery of the acarus will decide the question.

In addition to scabies, the fingers may be the seat of other cutaneous affections, such as lichen, psoriasis, tinea circinata, impetigo, dermatitis exfoliativa, pityriasis rubra, and the syphiliodermata. It is, however, hardly necessary to delay upon the resemblances which, according to some authorities, exist between them and eczema as sufficient has been said to point out the distinguishing features of eczema, so that with ordinary care a diagnosis may readily be made.

My practice in treating a patient with general or local eczema is (1) at once to relieve the portal circulation by mercurial and saline cathartics; to correct dyspeptic symptoms by acids or (as may be indicated) by alkalies, in some bitter vegetable infusion; to call the kidneys into functional activity by diuretics; to restore failing nerve energy by phosphorus; and to meet the gouty tendency by colchicum and alkalies, or the scrofulous by cod-liver oil and some preparation of iron. The acknowledged efficacy of certain natural waters—such as Harrogate, Strathpeffer, Aix-la-Chapelle, etc.—in chronic eczema, is doubtless due to their action upon the liver and the kidneys.

2. I pay particular attention to diet, limiting the albuminous and saccharine food, and forbidding the use of fermented liquors, and enforcing fresh fish and vegetable diet. Neglect of these and similar points often retard recovery in a very notable way.

3. Another subject to which I direct the patient's attention is that of *washing*. Usually I find that eczematous parts are washed much too frequently. It is an every day experience (especially among private patients) to hear a mother say that no matter how often she washes it she cannot keep her child's face clean of the scabs and crusts of eczema; while in truth, by her over zeal, she is frustrating the curative process of nature, which, until disturbed was proceeding beneath those crusts. As a rule it is best not to wash an eczema very often—certainly not more than once a day, sometimes not once a week—and then only for the purpose of removing the exfoliated epidermic scales and any ointments or applications which might otherwise irritate the skin. In such cases I do not recommend soaps; instead I advise oat-meal tea, or rain or soft water, to each pint of which one tablespoonful of glycerine has been added and two tablespoonfuls of finely-powdered starch.

4. Local treatment is doubtless the most important in eczema. In the acute forms it should consist of some bland, soothing fluid, or, if there be much exudation, some absorbent and slightly astringent powder. When the disease is more chronic, and all the heat and redness have given place to induration and scales, the latter should be removed by inunctions and poultices, and the former by stimulating applications. It is in the judicious employment of those remedies, and in the steady perseverance in their use when once decided upon, that such cases can be brought to a successful termination.

Reverting to the subject—that of local eczema of the fingers—the treatment I have found to answer best is:

(*a*). In the early or acute stage, while there is but little thickening, to apply on strips of old linen wrapped round each finger, and covered with a glove, either ung. hydrarg. ammoniat., of the strength of ʒ i ad ℥ i., or equal parts of vaseline and oleate of zinc (introduced by Mr. Crocker). If there be much tingling or itching I add to each ounce *m* x. of chloroform, or *m* xij. of acid hydrocyan. dil. The ointment should be allowed to remain undisturbed for about twenty-four hours. It should then be well washed off in oat-meal tea, and fresh ointment on fresh linen reapplied.

(*b*.) When induration exists, stronger and more stimulating ointments may be employed, or what will answer better at first, macerating the skin by a water poultice, or by simply covering the finger with an india rubber finger-stall. It should be closely fitting, but should not interfere with return of the venous blood. If the patients can wear these rubber finger-gloves, very rapid improvement will be seen to follow; the skin becomes pliable, soft, and thin, the scales disappear, and the rhagades close up, and the oleate of zinc ointment, or one made of bismuthi nitras (ʒ i ad ℥ i.) will speedily complete the cure.

Unfortunately, it is not every patient who will be persuaded to persist in the rubber treatment; their general objection is that the finger becomes so hot, and throbs so, that they either entirely desist from its use, or employ it so irregularly as to nullify all good effects. In such cases I have found Hebra's diachylon ointment, as modified by Kaposi, of the greatest service. Hebra was in the habit of treating indurated patches by rubbing in liq. potassæ till the thickened epidermis was dissolved, and the surface was covered with bleeding points; but I confess that in my experience, Irish patients will not submit to such treatment, for what reason I know not (except it be that their nervous system lies nearest the skin!), and that they who without a protest, submit to strong internal remedies producing considerable pain, will wince under and protest against remedies which would, when applied to the skin, cause slight pain and uneasiness.

For the itching, which is very severe in the stage of infiltration, there is no remedy so efficacious as tar and it may be employed either in the form of an alcoholic solution in combination with soap (saponis mol., ol. picis vel cadii, alcohol aa. partes æquales), or what I find answers better, in an ointment such as this: ℞ Cremoris frigid., ℥ i. ung, picis ʒ iij to ʒ vi, zinci oxidi ʒ i, hydrarg. ammoniat. ʒ j, vaselini ad ℥ ij. Ft. ung.

"Whatever course be adopted in treating chronic eczema, constancy and perseverance are of the most importance. He who is always changing his plan of treatment is sure not to attain his object so quickly as one who steadily and patiently applies whatever remedy seems best suited to the case." (Hebra.)

